# Clinical Outcomes Related to the After-Career Consultation in Retired Male Footballers

**DOI:** 10.1055/a-2684-8925

**Published:** 2025-09-03

**Authors:** Sean Carmody, Andrew Massey, Gino M Kerkhoffs, Vincent Gouttebarge

**Affiliations:** 1 26066 Department of Orthopedic Surgery and Sports Medicine, Amsterdam UMC location University of Amsterdam, Amsterdam, The Netherlands; 2 Amsterdam Collaboration for Health & Safety in Sports (ACHSS), IOC Research Center, Amsterdam, The Netherlands; 3 Medical Department, Fédération Internationale de Football Association (FIFA), Zurich, Switzerland; 4 Amsterdam Movement Sciences, Ageing & Vitality, Musculoskeletal Health, Sports, Amsterdam, The Netherlands; 5 Academic Center for Evidence based Sports medicine (ACES), Amsterdam, The Netherlands; 6 Football Players Worldwide (FIFPRO), Hoofddorp, The Netherlands; 7 Section Sports Medicine, University of Pretoria, Pretoria, South Africa

**Keywords:** player welfare, athlete health, long-term athlete care

## Abstract

The After-Career Consultation (ACC) was developed to empower the physical, mental and social health of retired professional footballers and effectively address their specific health challenges. The objective of this study was to describe the clinical outcomes (e.g., prevalence of health conditions) and recommendations to retired professional footballers who undertook the ACC. A quasi-experimental study was conducted. Forty-seven retired male professional footballers underwent ACCs. Ten participants had a diagnosis of osteoarthritis (21.3%), 4 (8.5%) met the criteria for a diagnosis of generalised anxiety disorder, 7 (14.9%) met the criteria for a diagnosis of depression and 7 (14.9%) met the criteria for Stage 1 hypertension. Health-related quality of life scores among retired footballers undergoing the ACC were above average compared to the general population. Clinical recommendations were made to participants in relation to their musculoskeletal ( *n* =12, 25.5%), cardiovascular ( *n* =12, 25.5%), mental ( *n* =10, 21.3%) and lifestyle ( *n* =20, 42.6%) health. Eleven participants (23.4%) were referred for further investigations, and secondary referral to other specialists was arranged for 4 (8.5%). Participants reported high satisfaction with the ACC. The ACC could complement existing player welfare strategies to provide a well-rounded approach to managing the long-term health of professional footballers throughout the lifespan.

## Introduction

There is an increasing awareness in professional sport of the need to provide medical after-care to retired athletes. 1​ 2​ ​3​ These initiatives stem from a recognition that although professional sport brings significant health benefits, some retired athletes may face specific health challenges related to their sporting career. 4​ In professional football, there is established evidence that retired players are at increased risk of musculoskeletal health issues in later life and that they may be more prone to mental health symptoms during the transition to retirement. 5​ ​6​ Common musculoskeletal health issues among retired professional footballers include early-onset osteoarthritis (OA), which is often associated with a history of severe injury and/or surgery during their playing career. 5​ ​7​ This, along with life events and lack of social support, may have a negative effect on the quality of life and/or mental health of retired professional footballers. 8​ 9​ ​10​ Additionally, there are emerging concerns that there may be an increased risk of neurocognitive conditions such as dementia among retired professional footballers. 11​ 12​ 13​ ​14​ This potentially increased risk is thought to relate to the effects of concussion and/or repetitive sub-concussive impacts (e.g., heading) on long-term neurological health, although more research is required to establish causal inference. 11​

In 2018, an After-Career Consultation (ACC) was developed to empower the physical, mental and social health of retired professional footballers and effectively address some of their specific health challenges. 15​ The ACC focussed on five health-related domains thought to be most relevant for retired players, namely detraining from professional football; management of OA; promotion of a healthy lifestyle; preventing mental and cognitive health problems; and employment and education. This intervention was well received by the retired professional footballers partaking in a pilot study. 15​ Therefore, Fédération Internationale de Football Association (FIFA) and Fédération Internationale des Associations de Footballeurs Professionnels (FIFPRO) agreed to collaborate from 2020 to provide retired male professional footballers worldwide with the ACC. 16​ The primary objective of this study was to describe the clinical outcomes (e.g., prevalence of health conditions, associations with contributing factors) and recommendations to retired male professional footballers who undertook the ACC. A secondary objective was to record the investigations and onward referrals arranged as a result of the ACC. A tertiary objective was to describe patient satisfaction and experience following the ACC.

## Methods

### Design

As randomisation was not practical or ethical, a quasi-experimental study based on a one-group post-test design was conducted, using the Transparent Reporting of Evaluations with Nonrandomized Designs (TREND) guidelines to ensure a high quality of reporting. 17​ ​18​ Ethical approval for the study was provided by the Medical Ethics Review Committee of the Amsterdam University Medical Centers, location University of Amsterdam (W21_135 # 21.150; Amsterdam, The Netherlands). The study was conducted in accordance with the principles set out in the Declaration of Helsinki (2013). 19​

### Participants

Between April 2021 and June 2023, a convenient sample of 47 retired male professional footballers underwent ACCs at FIFA Medical Centres (or a suitable equivalent) across six different countries. The inclusion criteria were: (i) male sex; (ii) retired professional footballer; (iii) ability to understand and speak English; and (iv) access to a clinic performing the ACC. In our study, a retired male professional footballer was defined as an individual who was remunerated for devoting several hours in all/most days (exceeding the time allocated to other types of professional or leisure activities) to playing football. 20​ Potential participants were informed by email and/or social media about the worldwide implementation of the ACC and related study via FIFPRO’s national player unions and via the individual network of the FIFA Medical Centres of Excellence. Retired players participated voluntarily in the study and did not receive any financial remuneration for their participation.

### Intervention

**Fig. 1:**
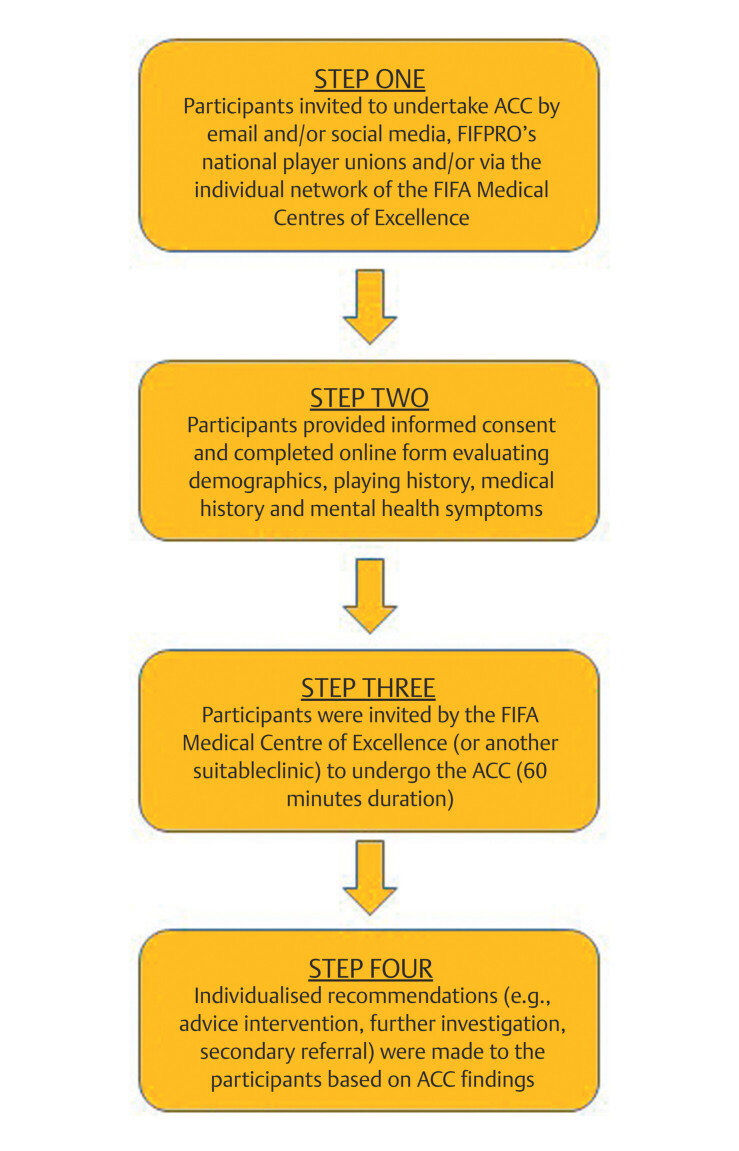
Flowchart detailing the patient journey for participants.

After giving their informed consent to partake in the study, participants completed an online form which evaluated, among other things, playing history, time elapsed since retirement, medical history, injury (and surgical) history, mental health symptoms and lifestyle factors. Upon completing the online form, the participants were invited by the FIFA Medical Centre of Excellence (or another suitable clinic) to undergo the ACC (60-min duration) at a convenient time by a suitably qualified physician. The ACC was individualised to each participant (based on their responses on the online form), but generally included an emphasis on musculoskeletal, mental, neurocognitive and cardiovascular health. Based on all information gathered through the ACC (self-reported and clinical findings), tailored recommendations (e.g., advice, intervention, further investigation, secondary referral) were made to the participants. A flowchart detailing the patient journey for participants is outlined in Fig. 1 . The information, registration, online, consultation and recommendation forms are available for review in the Supplementary material (available in the online version only). Completed forms were shared with and securely stored by the primary researcher.

### Primary outcome measures

Our primary outcome measures were related to the health conditions (and related recommendations) which have been identified as a concern among retired male professional footballers, related especially to the musculoskeletal health, mental health, neurocognitive health and cardiovascular health domains. 5​ The specific measurements for each domain are presented below.

#### Musculoskeletal health

The number of severe musculoskeletal injuries in the player’s career was recorded. A severe injury was defined as ‘an injury that occurred during team activities and led to either training or match absence for more than 28 days (4 wks)’. 21​ ​22​ The number of surgeries required as a result of an injury sustained during a footballer’s career was assessed by a single question; ‘How many surgeries as a consequence of an injury have you had during your professional football career?’. Clinical OA was assessed according to the definition provided by the National Institute for Health and Care Excellence, with the diagnostic criteria including: (i) the presence of activity-related joint pain, (ii) restricted range of motion and (iii) either no morning joint-related stiffness or morning stiffness that lasts no longer than 30 minutes. 23​

#### Mental health

Anxiety, depression, substance misuse and sleep disturbance were the mental health symptoms assessed during the ACC. Anxiety was assessed using the validated Generalised Anxiety Disorder (GAD-7) questionnaire, which is used to assess symptoms related to anxiety over the last 2 weeks (e.g., How often have you been bothered by not being able to stop or control worrying?). 24​ A total score ranging from 0 to 21 was calculated with higher sum scores indicating greater anxiety severity (0–4: normal; 5–9: mild; 10–14: moderate; and≥15: severe) and a sum score of≥10 indicating symptoms consistent with a diagnosis of generalised anxiety disorder. 20​ The Patient Health Questionnaire-9 was used to assess the presence of symptoms of depression in the previous 2 wks (e.g., ‘Have you been feeling down, depressed or hopeless?’) scored on a 4-point scale (from ‘not at all’ to ‘nearly every day’). A total sum score ranging from 0 to 27 was calculated, with higher sum scores indicating greater depression severity (0–4: normal; 5–9: mild; 10–14: moderate; and≥15: [moderate to] severe) and a sum score of≥10 indicating symptoms consistent with a diagnosis of depression. 25​ ​26​ The level of alcohol consumption was detected using the validated three-item Alcohol Use Disorders Identification Test (AUDIT-C; e.g., ‘How many standard drinks containing alcohol do you have on a typical day?’). A total score ranging from 0 to 12 was obtained by summing up the answers on the three items, with a score of 5 or more indicating the presence of alcohol misuse. 27​ ​28​ Sleep disturbance was measured using the shortened Athlete Sleep Screening Questionnaire (ASSQ), sleep disturbance in the previous recent past was assessed through five items (e.g., ‘How satisfied/dissatisfied are you with the quality of your sleep?’) scored on 4-point and 5-point scales. A total score ranging from 1 to 17 was obtained by summing up the answers to the five items, with a score of 8 or more indicating the presence of moderate sleep disturbance. 29​ ​30​

#### Lifestyle factors

Lifestyle factors assessed smoking status, diet and exercise. Smoking status was assessed through a single question (‘Do you smoke?’). Participants were invited to describe their diet, with five options ranging from ‘Very healthy’ to ‘Very unhealthy’. For exercise, participants were asked ‘In a typical week, on how many days do you do moderate - vigorous intensity sports, fitness or recreational (leisure) activities?’, with a free-text box provided to enter a number.

#### Health-related quality of life

The Patient-Reported Outcomes Measurement Information System Global Health (PROMIS-GH) was used to assess multiple domains related to health-related quality of life, such as physical health, levels of function, pain, social activities and fatigue. 31​ Based on 10 items, each measured on a 5-point scale (from 1 to 5) and subsequently converted, the Global Physical Health and Global Mental Health scores were calculated. 31​ The Global Physical Health score was calculated by adding the scores of items 3, 7, 9 and 10 of the quality of life questions on the questionnaire to obtain a subscale score out of 20. The questions relate to physical health, ability to carry out social activities and roles, fatigue and pain. The Global Mental Health score was calculated by adding the scores of items 2, 4, 5 and 8 to obtain a subscale score out of 20. The questions relate to quality of life, mental health, satisfaction with social activities and relationships and being bothered by emotional problems. Higher scores indicate a better quality of life. 31​

#### Neurocognitive health

A single question was asked related to concussion history; ‘How many concussions have you had during your professional football career?’. A concussion was defined as ‘a direct or transmitted blow to the head resulting in symptoms such as headache, nausea, vomiting, dizziness/balance problems, fatigue, trouble sleeping, drowsiness, sensitivity to light or noise, blurred vision, difficulty remembering, and difficulty concentrating’. 32​

#### Cardiovascular health

Cardiovascular health was assessed through screening questions (e.g., ‘Does the player experience relevant symptoms such as chest pain, palpitations, presyncope, syncope, shortness of breath?’), body composition (height, weight, skinfold assessment), blood pressure (BP), resting heart rate and a standard 12-lead electrocardiogram (ECG). BP was interpreted according to internationally accepted criteria (e.g.,≥140/90 mmHg indicates hypertension). 33​ ECG abnormalities were defined according to the international criteria for electrocardiographic interpretation in athletes. 34​

#### Clinical recommendations

Specific clinical recommendations were made to each player, dependent on the findings of the preceding assessment. In addition to the key medical domains highlighted above, the recommendations related to ‘lifestyle’ and ‘other’ are provided. If recommendations were made related to these domains, the physician completing the ACC was invited to highlight the degree of significance of these recommendations (e.g., slightly significant, significant, very significant). A free-text box was provided for the physician to elaborate on the recommendations they made based on the outcomes of the ACC.

### Secondary outcome measures

Our secondary outcome measures included the arrangement of further investigations and secondary referral based on the clinical findings of the ACC. These were assessed based on single questions (e.g., ‘Based on your assessment, do you plan to arrange any further investigation(s) for the retired footballer?’ and ‘Do you plan to arrange secondary referral for the retired footballer?’).

### Tertiary outcome measures

All players who underwent an ACC were contacted by email to complete a short (six-item) electronic survey evaluating their satisfaction and experience of the ACC. The survey included questions, such as ‘What is the primary reason(s) you choose to go to the After-Career Consultation?’, and statements, such as ‘Please indicate to what extent you agree with the following: I would recommend the After-Career Consultation to my (former) teammates with a health concern’ and ‘Do you have any suggestions to improve/alter the After-Career Consultation?’. Questions were answered on different response scales (e.g., ‘yes, no’ or free-text), while statements were scored on a 5-point Likert scale from ‘strongly disagree’ to ‘strongly agree’. The survey is available for review in the Supplementary material (available in the online version only).

### Descriptive variables

In addition to the outcomes stated above, the following descriptive variables were assessed: (family) history of cardiovascular disease, diabetes mellitus, medication use, employment status (post-retirement), professional football exposure (single questions about number of seasons and matches played), level of play, and educational level.

### Data analysis

All data analyses were conducted by using the statistical software IBM SPSS Statistics (Version 28.01.0). 35​ Descriptive analyses (mean, standard deviation, frequency, and range) were performed for all variables included in the study. For our primary objective, prevalence was calculated for all health conditions. Prevalence (expressed as a percentage) was calculated as the proportion of the number of participants with a given health condition relative to the total number of participants. Potential associations between contributing factors and independent variables were assessed using Spearman’s rank correlation coefficient. 36​ For our secondary and tertiary objectives, we used descriptive analyses (mean, standard deviation, frequency, and/or range) to evaluate the investigations and onward referrals arranged as a result of the ACC and present the participants’ satisfaction and experience of undergoing the ACC.

## Results

### Participant characteristics

Forty-seven retired male professional footballers underwent the ACC and were included for final analysis. The mean age of participants was 38.1 years (SD=5.4). The mean height and weight were 181.0 cm and 84.4 kg, respectively. The average duration of retirement at the time of the ACC was 5.5 years (SD=3.0). Twelve (25.5%) participants were from North America (CONCACAF) and 35 (74.5%) were from Europe (UEFA). The average number of seasons as a professional footballer was 15.8 years (SD=5.4). The majority ( *n* =43, 91.5%) reported primarily having played at the highest national level/league. Participants had played 390 games on average in their professional football career. Forty-two (89.4%) of the participants were in paid employment at the time of their ACC. Full participant characteristics are presented in Table 1 .

**Table:** **Table 1** Participant characteristics ( *n* =47)

Current age (yr)	38.1 mean, 5.4 SD, range 27–53
Height (cm)	181.0 mean, range 167.0–190.0
Weight (kg)	84.4, range 69.0–113.0
Nationality	
Italy	*n* =5, 10.6%
Malta	*n* =10, 21.3%
Mexico	*n* =10, 21.3%
Republic of Ireland	*n* =10, 21.3%
Spain	*n* =1, 2.1%
United Kingdom	*n* =9, 19.2%
USA	*n* =2, 4.3%
Currently in employment	
Yes	*n* =42, 89.4%
Number of years since retirement	5.47 (SD=3.0)
Number of seasons as a professional footballer	15.8 mean, range 1–30
Estimated no. of matches as a professional footballer	390 mean, range 2–1040
Played in the highest national league	*n* =43, 91.5%
Position (more than one option available for selection)	
Goalkeeper	*n* =7, 14.9%
Full back	*n* =7, 14.9%
Central defender	*n* =8, 17%
Defensive midfielder	*n* =6, 12.8%
Attacking midfielder	*n* =10, 21.3%
Winger	*n* =5, 10.6%
Striker	*n* =5, 10.6%

### Clinical outcomes and recommendations

#### Medical history

Eight of the participants (17%) reported a recent hospital admission for elective procedures (e.g., total hip replacement) or acute admission (e.g., renal colic, spontaneous pneumothorax). Five of the participants (10.6%) used regular medications (e.g., insulin, ADHD medication, asthma inhaler, PPI), with only one participant reporting regular use of non-steroidal anti-inflammatory medications. Five of the participants (10.6%) reported that they had been diagnosed with a condition relevant to their future health: including diabetes ( *n* =2), pericardial cyst, myocarditis, and psoriatic arthritis.

#### Musculoskeletal health

**Fig. 2:**
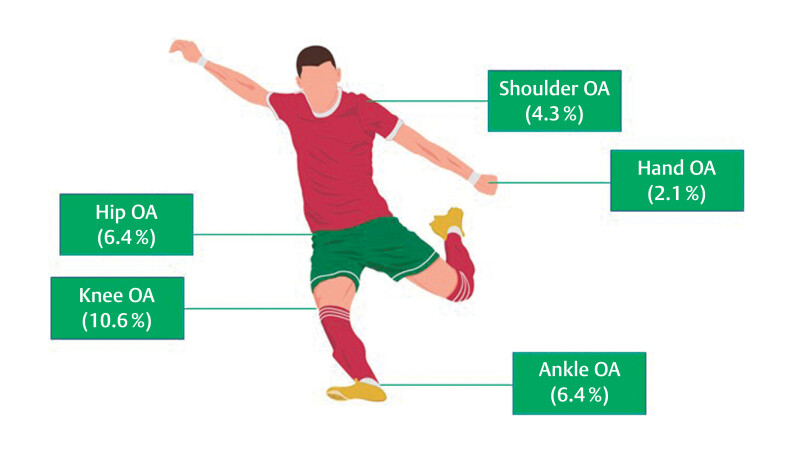
The distribution of osteoarthritis (OA) among participants.

A breakdown of the participants’ musculoskeletal health can be found in Table 2 . The average number of severe injuries (>4-wk time loss) sustained during their playing career was 3 per player (range 0–15). The average number of surgeries related to injuries sustained during their professional career was 2 per player (range 0–5). The prevalence of clinical OA (according to pre-defined criteria) 23​ was 21.3% ( *n* =10). The average age of the participants with evidence of clinical OA was 39.1 yrs (range 34–53). The distribution of clinical OA in these 10 participants is shown in Fig. 2 .

**Table:** **Table 2** Medical conditions ( *n* =47)

Musculoskeletal health ( *n* =46)	
No. of severe injuries	3.2 mean, SD=3.4 (range 0–15)
No. of surgeries	1.6 mean, SD=1.6 (range 0–5)
Mental health of participants ( *n* =47)	
GAD-7	4.3 mean (SD=4.2)
PHQ-9	4.0 mean (SD=5.0)
AUDIT-C	3.5 mean (SD=1.9)
Lifestyle factors ( *n* =47)	
ASSQ	5.4 mean (SD=2.6)
Smoker	*n* =2, 4.3%
Health-related quality of life	
Global Physical Health score mean	39.8
Global Mental Health score mean	41.7
Neurocognitive health ( *n* =45)	
No. of participants who had been diagnosed with a concussion	*n* =13
Mean no. of concussions per participant	0.4
Mean no. of concussions per participant diagnosed with a concussion	2 (range 1–4)
Cardiovascular health ( *n* =46)	
Abnormal ECG findings	*n* =5, 10.9%

GAD-7, General Anxiety Disorder 7 (0–4: normal; 5–9: mild; 10–14: moderate; and≥15: severe); PHQ-9, Patient Health Questionnaire-9 (0–4: normal; 5–9: mild; 10–14: moderate; and≥15: [moderate to] severe); AUDIT-C, alcohol use disorders identification test consumption (a score of 5 or more indicating the presence of alcohol misuse); ASSQ, Athlete Sleep Screening Questionnaire (a score of 8 or more indicating the presence of moderate sleep disturbance); ECG, electrocardiogram.

#### Mental health

The 2-wk prevalence of symptoms consistent with a diagnosis of generalised anxiety disorder was 8.5% ( *n* =4), with 29.8% ( *n* =14) meeting criteria for mild anxiety. The 2-wk prevalence of symptoms consistent with a diagnosis of depression was 14.9% ( *n* =7), including two individuals with moderate–severe depression. Five participants (10.6%) showed evidence of mild depression. Nearly a quarter ( *n* =11) misused alcohol. Five participants (10.6%) had evidence of moderate sleep disturbance, with two (4.3%) meeting the criteria for severe sleep disturbance. Data on the mental health of the participants are presented in Table 2 .

#### Lifestyle factors

Two of the participants were smokers (4.3%). Over half ( *n* =25) of the participants reported their diet to be either ‘very healthy’ ( *n* =7, 14.9%) or ‘healthy’ ( *n* =18, 38.3%). In total, participants reported engaging in moderate to vigorous exercise (e.g., sports, recreational activities, fitness) on 3 days per week on average. Data on lifestyle factors of the participants are presented in Table 2 .

#### Health-related quality of life

The mean Global Physical Health score among retired professional footballers was 51.9. The mean Global Mental Health score among retired professional footballers was 51.7 (see Table 2 ).

#### Neurocognitive health

Thirteen (27.7%) of the participants reported being diagnosed with a concussion during their career. For those who had been diagnosed with a concussion, the average was 2 concussions per player (range 1–4) (see Table 2 ).

#### Cardiovascular health

Seven participants (14.9%) met the criteria for Stage 1 hypertension; namely, a clinic BP reading ranging from 140/90 to 159/99 mmHg. 37​ Forty-six participants underwent a 12-lead ECG, from which five participants (10.9%) had an abnormal ECG finding according to pre-defined criteria. 34​ Abnormal ECG findings among these five participants included anterior ( *n* =2) and inferior t-wave inversion ( *n* =1), features of left atrial enlargement ( *n* =2), and complete right bundle branch block ( *n* =1). Data on the cardiovascular health of the participants are presented in Table 2 .

#### Clinical recommendations

Clinical recommendations were made to 12 (25.5%) participants in relation to their musculoskeletal health. Half of these findings/recommendations were deemed by the clinician to be ‘significant’ (e.g., arrange magnetic resonance imaging [MRI] of both knees, limit weight-bearing exercise) and ‘very significant’ (e.g., total hip replacement likely needed due to OA). Clinical recommendations were made to 12 (25.5%) of participants in relation to their cardiovascular health, with these findings/recommendations thought to be ‘significant’ in five of the cases (e.g., annual cardiology review) and ‘slightly significant’ in the remainder (e.g., regular BP monitoring). Clinical recommendations were only made to one player in relation to neurocognitive health, which was deemed to be ‘significant’ (arrange psychiatric review in relation to a pre-existing diagnosis of ADHD and associated sleep, mood and substance misuse issues). Ten players (21.3%) received recommendations in relation to their mental health, with two of these recommendations highlighted as ‘very significant’ (e.g., psychiatric review). Twenty participants (42.6%) received lifestyle recommendations, with nine deemed ‘slightly significant’ (e.g., maintain a healthy diet) and 8 ‘significant’ (17.0%) (e.g., reduce alcohol intake, improve sleep habits). No additional recommendations were made to any participants who were in health domains separate from those already described.

### Investigations and referrals

Eleven of the participants (23.4%) were referred for further investigations based on the findings from their ACC. These investigations included blood tests, urine tests, ambulatory BP monitoring, cardiac investigations (e.g., echocardiogram) and imaging (e.g., MRI). For 12 participants (25.5%), the ACC clinician arranged follow-up, and where specified, this ranged from follow-up in 1 week (e.g., for significant mental health issues) to 6 months (e.g., repeat routine cardiac investigations, OA surveillance monitoring). Secondary referral was arranged in four cases (8.5%).

### Satisfaction and experience

Twenty-four (51.0%) of participants completed the short (six-item) electronic survey evaluating their satisfaction and experience of the ACC. The primary reasons for participants undertaking the ACC were due to a physical health concern (e.g., past injury, joint pain) ( *n* =4, 17%), a mental health concern ( *n* =1, 4.2%), a general health check-up ( *n* =11, 45.8%), to support the scientific advancement and understanding of the long-term health of footballers ( *n* =12, 50%). Most participants ( *n* =22, 91.7%) agreed or strongly agreed that the ACC met their expectations. Nineteen participants (79.2%) ‘strongly agree’ with the statement ‘I would recommend the After-Career Consultation to my (former) teammates with a health concern’. Twenty-two participants (91.7%) agreed or strongly agreed that the ACC adds value to the medical provision received during their football career. Participants had a ‘free-text’ option to provide suggestions to improve/alter the ACC. Seven participants offered suggestions, and these included integrating routine blood tests into the ACC, a request to offer more detailed mental health support and to arrange follow-up every 5 years. One participant felt that more assistance should have been provided for the issues highlighted during the ACC. Satisfaction/experience of the ACC is presented in Table 3 .

**Table:** **Table 3** Satisfaction and experience ( *n* =24, 51%)

Primary reason(s) to choose to go to the ACC (up to three reasons permitted)	
Physical health concern (e.g., past injury, joint pain)	*n* =4 (17%)
Mental health concern (e.g., sleep problem)	*n* =1 (4%)
Brain health concern (e.g., previous concussion)	*n* =0 (0%)
Heart health concern (e.g., blood pressure)	*n* =0 (0%)
General health check-up	*n* =11 (46%)
To support the scientific advancement and understanding of the long-term health of footballers	*n* =12 (50%)
Perception and satisfaction with ACC (%)	
The ACC met the expectations of the participants	92
The ACC adds value to the medical provision received during their football career	92
Participants would recommend the ACC to ex-players **without** a health concern	92
Participants would recommend the ACC to ex-players **with** a health concern	92

## Discussion

This study described the clinical outcomes of and recommendations to retired male professional footballers who undertook the ACC between April 2021 and June 2023. The prevalence of a range of health conditions was identified (e.g., clinical OA=21%, anxiety/depression=15%, hypertension=15%) with subsequent recommendations and referrals recorded. Participants reported high satisfaction with the ACC, and the primary reason (50%) for attending the ACC was to support the scientific advancement and understanding of the long-term health of footballers.

### Health conditions among retired professional footballers

According to current epidemiological evidence, all-cause mortality is lower among retired male professional footballers than among matched controls, with fewer comorbidities such as diabetes and cancer. 5​ ​11​ The prevalence of health conditions varies across the lifespan of a footballer, with mental health issues presenting during periods of adjustment (e.g., de-selection, injury, transition to retirement), 38​ 12​ ​40​ significant musculoskeletal issues typically presenting earlier compared to the general population (e.g., aged 45 onwards), 5​ and neurocognitive issues emerging after the age of 65–70. 11​ ​12​ Within the demographic of retired male professional footballers in this study (mean age 38 yrs) the common health conditions identified included musculoskeletal, mental health and cardiovascular health conditions, with no neurocognitive health conditions identified. These findings may reflect the age profile, proximity to retirement, and injury history of the former players included in our study.

Our study found a prevalence of 11% of knee OA, which is low when compared to other studies among retired male professional footballers (range 9–80%). 6​ The prevalence of hip OA was 6%, which aligns with other studies, where hip OA ranges from 2 to 14%, although it has been reported as high as 50%. 6​ ​41​ Ankle OA ranges from 4 to 35% among retired male professional footballers, 42​ ​43​ with a prevalence of 6% in our study. The method used to diagnose OA in various limb joints differs across studies, with some relying on clinical assessment, imaging findings, or self-reporting by study participants—or in the case of our study, a combination of methods (clinical assessment and self-reporting). The broad-ranging differences in findings between studies highlight the need for consistency in data collection in order to make valid comparisons and accurate health recommendations.

Depression, anxiety, sleep disturbance, and alcohol misuse were the mental health issues investigated among retired male professional footballers in our study. The prevalence of symptoms consistent with a diagnosis of generalised anxiety disorder was 9% ( *n* =4), with 30% ( *n* =14) meeting criteria for mild anxiety. The prevalence of symptoms consistent with a diagnosis of depression was 15% ( *n* =7), including two individuals with moderate–severe depression. Five participants (11%) showed evidence of mild depression. These findings are similar to those seen in a study by Fernandes et al., where prevalence of depression and anxiety was reported in 6 and 12% of retired male professional footballers, 39​ but considerably lower than rates of prevalence seen in other studies of retired male professional footballers (35–39%) which may relate to the use of different validated instruments to assess anxiety/depression in this population. 44​ ​45​ Nearly a quarter of participants ( *n* =11) misused alcohol in our study, which is similar to a study by Gouttebarge et al. (18%). 45​ A recent study among male ( *n* =81; mean age of 39 yrs; mean career duration of 12 yrs) retired professional footballers from the Australian league showed prevalence rates ranging from 11% for anxiety to as high as 69% for alcohol misuse, which demonstrates that there may be region-specific and socioeconomic considerations among retired professional footballers. 46​

No studies, to our knowledge, have specifically examined the prevalence of cardiovascular health conditions among retired male professional footballers. 6​ Our study identified hypertension among seven participants (15%), while five participants (11%) had an abnormal ECG finding according to pre-defined criteria. There are studies that have indirectly examined issues related to cardiovascular health in retired professional footballers. Two studies highlighted that retired footballers were more likely than current footballers to adopt behaviours which may increase their risk of cardiovascular disease, for example, smoking, alcohol misuse and poor nutritional behaviours. 41​ ​47​ The prevalence of smoking in our study (4%) was lower than that seen in the general population (13%). 48​ Until such time as there is a better understanding of cardiovascular health outcomes among retired professional footballers, population-wide clinical guidelines for the management of cardiovascular health conditions should be adopted.

#### Neurocognitive health concerns among retired professional footballers

Thirteen (28%) of the participants reported being diagnosed with a concussion during their career (average 2 concussions per player; range 1–4). Several studies have highlighted the concerns related to the potential increased risks of neurocognitive disorders among retired male professional footballers, and this may be related to a history of concussion and/or repeated sub-concussive impacts, although more research is required to demonstrate causality. 11​ 12​ 13​ ​14​ While neurocognitive function was not specifically assessed during the ACC, there were no new significant neurocognitive concerns highlighted among the footballers, with recommendations only made to one participant in relation to his neurocognitive health (this was related to a pre-existing psychiatric condition). The likely explanation for this is that the mean age of participants was 38 years, with the mean age of retired football players in the study by *Macnab* et al. 64 years. 12​ Nevertheless, the ACC identified several modifiable contributing factors for neurocognitive disorders among the retired players, such as smoking 49​ (4%), excessive alcohol consumption 50​ (23%) and depression (15%). 51​ The ACC can be used as an opportunity to address modifiable contributing factors for neurocognitive disorders among retired professional footballers, as has been done in other contexts. 3​ It may be appropriate to risk stratify retired players based on their lifestyle behaviours, history of concussion(s), and/or mental health disorders 52​ and consider targeted evidence-based interventions for those identified as being at higher risk due to the aforementioned contributing factors.

#### Future directions

The retired professional footballers in this study expressed high satisfaction rates with the ACC, similar to other after-care initiatives offered to professional footballers. 53​ Initiatives such as the ACC should complement existing efforts to improve the welfare of current, future, and former professional footballers. These efforts include, but are not limited to, effective injury prevention methods, improving concussion management, developing mental health literacy, monitoring player loads, and appropriate surgical decision-making. 6​ ​53​ Rather than a once-off assessment, serial follow-ups (e.g., every 5 yr) of the ACC may prove more beneficial to retired professional footballers—as one of the participants proposed. There are very few studies examining health outcomes among retired women’s professional footballers, and with the rapid development of the women’s game, this should be a priority. Accordingly, in addition to the health domains of musculoskeletal, mental, neurocognitive and cardiovascular health, the ACC should focus on a female-specific domain and explore reproductive health outcomes among retired women’s professional footballers. 54​ Expanding the geographical scope of interventions such as the ACC (or similar) across multiple sports is likely to further benefit long-term athlete welfare. Finally, considering the number of years since retirement for future ACCs is important, given the pattern of health complaints presenting at different times across the lifespan of a footballer, and the difference in resources and conditions that existed for players of different generations.

#### Strengths and limitations

This study is the first to investigate the effect of the ACC across multiple countries and continents. The small sample size is the main limitation of this study, which impacts the generalisability of the findings. It is also limited by its use of the English language only during the course of the ACC, and its findings are limited by not including participants from continents outside of Europe or North America. The non-randomised nature of this study increases risk of bias, confounding and validity issues. In particular, the risk of selection bias in this study may lead to an over-estimation of certain health conditions among retired male professional footballers. There was no reference or comparison group from a non-athlete population, matched for age and gender, and this would have allowed for a greater understanding of potential differences among retired male professional footballers and those in the general population. Despite the challenges of doing so, carrying out a randomised controlled trial (RCT) would be the most effective method of understanding the true effect of retired professional footballers undertaking the ACC.

## Conclusion

This study described the clinical outcomes/recommendations for retired male professional footballers who undertook the ACC. Participants reported high satisfaction with the ACC. The ACC could complement existing player welfare strategies (e.g., concussion care, load management) to provide a well-rounded approach to managing the long-term health of professional footballers during and after their careers. Future studies should assess the impact of the intervention against a control group, and among women footballers.

## Disclosure statement

The authors declare no relevant financial or non-financial competing interests.

## Data availability statement

The data that support the findings of this study are available on request from the corresponding author.

## Supplementary Material

Supplementary Material Zusatzmaterial
