# Supplementary material for: Clinical Outcomes Related to the After-Career Consultation in Retired Male Footballers
Source: Int J Sports Med. 2025 Sep 3;47(3):226–34. doi: 10.1055/a-2684-8925 (PMC12948490; doi:10.1055/a-2684-8925)
Supplement: Supplementary file 1 — Supplementary Material [file 10-1055-a-2684-8925_26874943.pdf]

Thieme

AFTER CAREER CONSULTATION IN PROFESSIONAL FOOTBALL

Registration form

Thank you for taking the time to complete this Registration Form in relation to the After Career Consultation (ACC) for Retired Professional Footballers. The aim of the ACC is primarily to support your health during retirement, but also to understand the wider health issues experienced by retired footballers.

If you need any further information regarding the ACC, please contact:

<insert email address>

Please complete the following form in order to register for the After Career Consultation.

1. First name

2. Last name

3. Date of birth

4. Age

5. Nationality

6. Languages spoken

7. Email address

8. Street name and number

9. ZIP/Postal code

10. City

11. Primary Care Physician name and address (enter ‘N/A’ if none)

12. Consent

To complete your registration, we kindly read the following and tick all boxes to proceed:

- I have been fully informed about the After Career Consultation and the related study. I understand that I have the right to withdraw from the ACC process at any point without disclosing my reasons.

- I consent to the collection and confidential use of my data in order to answer all questions of the After Career Consultation and related study.

- I consent to my medical data being shared with my Primary Care Physician (where appropriate)

Int J Sports Med | DOI 10.1055/a-2684-8925 | © Thieme. All rights reserved.

- I would like to proceed with the After Career Consultation.

- I would like to participate in the related study in order to help better understand the health issues experienced by retired footballers.

Int J Sports Med | DOI 10.1055/a-2684-8925 | © Thieme. All rights reserved.

AFTER CAREER CONSULTATION IN PROFESSIONAL FOOTBALL

Online form

The aim of the following online questionnaire is to explore your playing history and medical history - completing this will help you to get the most out of your After Career Consultation.

For some questions, you can choose an answer from multiple options by checking a box. It is important that you choose the most appropriate answer for yourself. There are also some questions that require you to fill in the answer on the provided space. Please answer all the questions.

1. Characteristics

1.1 For how many seasons were you a professional footballer?

____

1.2 How many matches did you play in professional football?

____

1.3 At what level did you mainly play?
- Highest national level/league
- Second highest national level/league
- Other

1.4 What is your main position on the field?
- Goalkeeper
- Full back
- Central defender
- Defensive midfielder
- Attacking midfielder
- Winger
- Striker

1.5 What was your dominant leg as player?
- Right
- Left

1.6 How long have you been retired from professional football?

____

1.7 What is the highest level of education you have completed?
- No school completed
- Nursery/Elementary school

Int J Sports Med | DOI 10.1055/a-2684-8925 | © Thieme. All rights reserved.

- High school
- Vocational/technical school
- College, university or equivalent

1.8 Are you currently studying?
- Yes
- No

1.9 Do you have currently another paid job?
- Yes
- No

If yes, what kind of (paid) work do you do?

____

If yes, how many hours per week on average do you work?

____

1.10 What is your current civil/marital status?
- Single
- In a relationship
- Married
- Divorced/separated
- Widowed

2. Medical history

2.1 In the past few years, were you admitted to a hospital?
- Yes
- No

If yes, why?

____
____
____

2.2 Do you use medications?
- Yes
- No

If yes, which one(s)?

____
____
____

2.3 Have you been diagnosed with a disease or illness relevant for your future health?

Int J Sports Med | DOI 10.1055/a-2684-8925 | © Thieme. All rights reserved.

- Yes
- No

If yes, which one(s)?

2.4 Has an immediate family member been diagnosed with a disease or illness relevant for your own future health?

- Yes
- No

If yes, which one(s)?

3. Severe injuries, surgeries and concussions

3.1 How many severe injuries have you had during your professional football career?
A severe injury is an injury that occurred during team activities and led to either training or match absence for more than 28 days (4 weeks).

3.2 How many surgeries as a consequence of an injury have you had during your professional football career?

3.3 How many concussions have you had during your professional football career?
A concussion is a direct or transmitted blow to the head resulting in symptoms such as headache, nausea, vomiting, dizziness/balance problems, fatigue, trouble sleeping, drowsiness, sensitivity to light or noise, blurred vision, difficulty remembering, and difficulty concentrating.

4. Mental health

Int J Sports Med | DOI 10.1055/a-2684-8925 | © Thieme. All rights reserved.

4.1 The following questions relate to feeling anxious or stressed. Over the last 2 weeks, how often have you been bothered by the following problems?

	Not at all	Several days	More than half the days	Nearly every day
1. Feeling nervous, anxious, or on edge				
2. Not being able to stop or control worrying				
3. Worrying too much about different things				
4. Trouble relaxing				
5. Being so restless that it's hard to sit still				
6. Becoming easily annoyed or irritable				
7. Feeling afraid as if something awful might happen				

4.2 The following questions relate to feeling depressed, sad or blue. Over the past 2 weeks, how often have you been bothered by any of the following problems?

	Not at all	Several days	More than half the days	Nearly every day
1. Little interest or pleasure in doing things				
2. Feeling down, depressed or hopeless				
3. Trouble falling asleep, staying asleep, or sleeping too much				
4. Feeling tired or having little energy				
5. Poor appetite or overeating				
6. Feeling bad about yourself - or that you're a failure or have let yourself or your family down				
7. Trouble concentrating on things, such as reading the newspaper or watching television				
8. Moving or speaking so slowly that other people could have noticed. Or, the opposite - being so fidgety or restless that you have been moving around a lot more than usual				
9. Thoughts that you would be better off dead or of hurting yourself in some way				

4.3 The following questions relate to your sleep habits. Please give the best answer which you think represents your typical sleep habits over the recent past.

1. How many hours of actual sleep do you get at night? (This may be different than the number of hours you spent in bed.)
    - 5 to 6 hours
    - 6 to 7 hours
    - 7 to 8 hours
    - 8 to 9 hours
    - more than 9 hours
2. How satisfied/dissatisfied are you with the quality of your sleep?
    - Very satisfied
    - Somewhat satisfied
    - Neither satisfied or dissatisfied
    - Somewhat dissatisfied
    - Very dissatisfied
3. How long does it usually take you to fall asleep each night?
    - 15 minutes or less
    - 16 – 30 minutes
    - 31 – 60 minutes

Int J Sports Med | DOI 10.1055/a-2684-8925 | © Thieme. All rights reserved.

- Longer than 60 minutes

4. How often do you have trouble staying asleep?
    - Never
    - Once or twice per week
    - Three or four times per week
    - Five to seven days per week

5. How often do you take medicine to help you sleep (prescribed or over-the-counter)?
    - Never
    - Once or twice per week
    - Three or four times per week
    - Five to seven days per week

5. Life style

5.1 In a typical week, on how many days do you do moderate - vigorous intensity sports, fitness or recreational (leisure) activities?

____
____
____

5.2 Do you smoke?
- Yes
- No

5.3 How often do you have a drink containing alcohol?
- ☐ Never
- ☐ Monthly or less
- ☐ 2-4 times a month
- ☐ 2-3 times a week
- ☐ 4 or more times a week

5.4 How many standard drinks containing alcohol do you have on a typical day?
- ☐ None or 1 or 2
- ☐ 3 or 4
- ☐ 5 or 6
- ☐ 7, 8 or 9
- ☐ 10 or more

5.5 How often do you have 6 or more drinks containing alcohol on one occasion?
- ☐ Never
- ☐ Less than monthly
- ☐ 2-4 times per month
- ☐ 2-3 times per week
- ☐ 4 or more times per week

5.6 In general, how would you describe your diet?

- ☐ Very healthy
- ☐ Healthy
- ☐ Adequate

Int J Sports Med | DOI 10.1055/a-2684-8925 | © Thieme. All rights reserved.

- Unhealthy
- Very unhealthy

5.7 In general, would you say your health is:
- Excellent
- Very good
- Good
- Fair
- Poor

5.8 In general, would you say your quality of life is:
- Excellent
- Very good
- Good
- Fair
- Poor

5.9 In general, how would you rate your physical health?
- Excellent
- Very good
- Good
- Fair
- Poor

5.10 In general, how would you rate your mental health, including your mood and your ability to think?
- Excellent
- Very good
- Good
- Fair
- Poor

5.11 In general, how would you rate your satisfaction with your social activities and relationships?
- Excellent
- Very good
- Good
- Fair
- Poor

5.12 In general, please rate how well you carry out your usual social activities and roles. (This includes activities at home, at work and in your community, and responsibilities as a parent, child, spouse, employee, friend, etc.).
- Excellent
- Very good
- Good
- Fair
- Poor

5.13 To what extent are you able to carry out your everyday physical activities such as walking, climbing stairs, carrying groceries, or moving a chair?
- Completely
- Mostly

Int J Sports Med | DOI 10.1055/a-2684-8925 | © Thieme. All rights reserved.

☐ Moderately
☐ A little
☐ Not at all

5.14 In the past 7 days, how often have you been bothered by emotional problems such as feeling anxious, depressed or irritable?

☐ Never
☐ Rarely
☐ Sometimes
☐ Often
☐ Always

5.15 In the past 7 days, how would you rate your fatigue on average?

☐ None
☐ Mild
☐ Moderate
☐ Severe
☐ Very severe

5.16 In the past 7 days, how would you rate your pain on average?

☐ ☐ ☐ ☐ ☐ ☐ ☐ ☐ ☐ ☐ ☐
0 10
No pain Worst pain imaginable

Int J Sports Med | DOI 10.1055/a-2684-8925 | © Thieme. All rights reserved.

AFTER CAREER CONSULTATION IN PROFESSIONAL FOOTBALL

Consultation form

Date of Consultation

Key Summary Based on Online Form:

Name:

DoB (Age):

Severe injuries:

Previous concussion(s):

Previously diagnosed medical problems:

Previously diagnosed mental Health Issues:

Medications:

Other:

Consultation:

- Review any significant issues identified in the online form
- Explore the ideas, concerns and expectations of the retired footballer

Int J Sports Med | DOI 10.1055/a-2684-8925 | © Thieme. All rights reserved.

Clinical examination

1. Height (cm)

2. Weight (kg)

3. Percentage of body fat (skinfold thickness measured on 4 sites: biceps, triceps, subscapular, suprailiac)

Cardiovascular Assessment

4. Blood pressure:

5. Electrocardiogram abnormalities
A standard resting 12-lead electrocardiogram (ECG) is recorded (not directly after physical activity) with the participant in the supine position during quiet respiration and recorded at 25mm/s with a gain setting of 10mm/mV.
5.1 Date of the ECG
5.2 Resting heart rate
5.3 Does the player experience relevant symptoms such as chest pain, palpitations, presyncope, syncope, shortness of breath?
5.4 Did you observe any abnormal and/or borderline ECG findings?

•	**No abnormalities observed and normal ECG findings in (former) athletes**
•	**T wave inversion**<br>≥1 mm in depth in two or more contiguous leads; excludes leads aVR, III and V1<br>Anterior > V2-V4 (excluding black athletes with J-point elevation and convex ST segment elevation followed by TWI in V2-V4; athletes < age 16 with TWI in V1-V3; and biphasic T waves in only V3)
•	**T wave inversion**<br>≥1 mm in depth in two or more contiguous leads; excludes leads aVR, III and V1<br>Lateral > I and AVL, V5 and/or V6 (only one lead of TWI required in V5 or V6)
•	**T wave inversion**<br>≥1 mm in depth in two or more contiguous leads; excludes leads aVR, III and V1<br>Inferolateral > II and aVF, V5-V6, I and AVL
•	**T wave inversion**<br>≥1 mm in depth in two or more contiguous leads; excludes leads aVR, III and V1<br>Inferior > II and aVF
•	**ST segment depression**<br>≥0.5 mm in depth in two or more contiguous leads
•	**Pathological Q waves**<br>Q/R ratio ≥0.25 or ≥40 ms in duration in two or more leads (excluding III and aVR)
•	**Complete left bundle branch block**<br>QRS ≥120 ms, predominantly negative QRS complex in lead V1 (QS or rS) and upright notched or slurred R wave in leads I and V6
•	**Profound non-specific intraventricular conduction delay**<br>Any QRS duration ≥140 ms

Int J Sports Med | DOI 10.1055/a-2684-8925 | © Thieme. All rights reserved.

•	**Epsilon wave**<br>Distinct low amplitude signal (small positive deflection or notch) between the end of the QRS complex and onset of the T wave in leads V1-V3
•	**Ventricular pre-excitation**<br>PR interval <120 ms with a delta wave (slurred upstroke in the QRS complex) and wide QRS (≥120 ms)
•	**Prolonged QT interval**<br>QTc ≥470 ms (male)<br>QTc ≥480 ms (female)<br>QTc ≥500 ms (marked QT prolongation)
•	**Brugada type 1 pattern**<br>Coved pattern: initial ST elevation ≥2 mm (high take-off) with downsloping ST segment elevation followed by a negative symmetric T wave in ≥1 leads in V1-V3
•	**Profound sinus bradycardia**<br><30 beats per minute or sinus pauses ≥3 s
•	**Profound 1° atrioventricular block**<br>≥400 ms
•	**Mobitz type II 2° atrioventricular block**<br>Intermittently non-conducted P waves with a fixed PR interval
•	**3° atrioventricular block**<br>Complete heart block
•	**Atrial tachyarrhythmias**<br>Supraventricular tachycardia, atrial fibrillation, atrial flutter
•	**Premature ventricular contractions**<br>≥2 premature ventricular contractions per 10 s tracing
•	**Ventricular arrhythmias**<br>Couplets, triplets and non-sustained ventricular tachycardia
•	**Left axis deviation**<br>−30° to −90°
•	**Left atrial enlargement**<br>Prolonged P wave duration of >120 ms in leads I or II with negative portion of the P wave ≥1 mm in depth and ≥40 ms in duration in lead V1
•	**Right axis deviation**<br>>120°
•	**Right atrial enlargement**<br>P wave ≥2.5 mm in II, III or aVF
•	**Complete right bundle branch block**<br>rSR′ pattern in lead V1 and an S wave wider than R wave in lead V6 with QRS duration ≥120 ms

Musculoskeletal Assessment

6. Clinical osteoarthritis

Please conduct a history and physical examination in order to assess the presence / absence of clinical osteoarthritis in both ankles, both knees and both hips.<br><br>Clinical osteoarthritis is diagnosed when:<br><br>1. activity-related joint pain is present,<br><br><u>AND</u> 2. range of motion of the joint is restricted,<br><br><u>AND</u> 3. either no morning joint-related stiffness or morning stiffness that lasts no longer than 30 minutes.

6.1 Date of clinical osteoarthritis assessment

6.2 Clinical diagnosis of osteoarthritis
- No osteoarthritis
- Left ankle
- Right ankle

Int J Sports Med | DOI 10.1055/a-2684-8925 | © Thieme. All rights reserved.

- Left knee
- Right knee
- Left hip
- Right hip
- Other namely:

____
____
____

Mental Health Assessment

7.1

	Total score	Threshold
Anxiety: calculate the total score by summing up the answers on the 7 items from 4.1 (online form)		≥ 10
Depression: calculate the total score by summing up the answers on the 9 items from 4.2 (online form)		≥ 10
Sleep disturbance: calculate the total score by summing up the answers on the 5 items from 4.3 (online form)		≥ 8
Alcohol misuse disturbance: calculate the total score by summing up the answers on the 3 items from 5.3, 5.4 and 5.5 (online form)		≥ 4
In case that one or more total scores are reaching a given threshold, consider actioning the following:<br><br>1. conduct a comprehensive clinical assessment,<br><br>AND 2. identify significant problems/diagnoses<br><br>AND 3. discuss and create a management/intervention plan (refer to the International Olympic Committee consensus statement on mental health in elite athletes for guidance)<br><br>AND 4. Consider a referral to a mental health professional (e.g., clinical psychologist or psychiatrist).		

7.2 Has a significant mental health problem been identified?

- Yes
- No

If yes, please provide a suspected diagnosis below:

____
____
____

Int J Sports Med | DOI 10.1055/a-2684-8925 | © Thieme. All rights reserved.

8.1 Further Assessment

Please document the findings from any further clinical assessment performed below

Int J Sports Med | DOI 10.1055/a-2684-8925 | © Thieme. All rights reserved.

AFTER CAREER CONSULTATION IN PROFESSIONAL FOOTBALL

Recommendation form

1. Musculoskeletal health
Based on the information gathered during the After Career Consultation (medical history, online form, examination), are there any recommendations provided to the player with regard to his musculoskeletal health?
- Yes
- No

If yes, how would you rate these recommendations?
- Slightly significant
- Significant
- Very significant

If yes, which recommendations did you make?

2. Cardiovascular health
Based on the information gathered during the After Career Consultation (medical history, online form, examination), are there any recommendations provided to the player with regard to his cardiovascular health?
- Yes
- No

If yes, how would you rate these recommendations?
- Slightly significant
- Significant
- Very significant

If yes, which recommendations did you make?

3. Neurological OR Neurocognitive health
Based on the information gathered during the After Career Consultation (medical history, online form, examination), are there any recommendations provided to the player with regard to his neurocognitive health?
- Yes
- No

If yes, how would you rate these recommendations?
- Slightly significant
- Significant
- Very significant

If yes, which recommendations did you make?

Int J Sports Med | DOI 10.1055/a-2684-8925 | © Thieme. All rights reserved.

4. Mental health
Based on the information gathered during the After Career Consultation (online form, consultation), are there any recommendations provided to the player with regard to his mental health?

- Yes
- No

If yes, how would you rate these recommendations?

- Slightly significant
- Significant
- Very significant

If yes, which recommendations, interventions or strategies did you make?

7.3 Did you refer the player to a mental health professional (e.g., clinical psychologist or psychiatrist)?

- No
- Yes

If yes, please provide details below:

5. Lifestyle
Based on the information gathered during the After Career Consultation (medical history, online form, examination), are there any recommendations provided to the player with regard to his lifestyle?

- Yes
- No

If yes, how would you rate these recommendations?

- Slightly significant
- Significant
- Very significant

If yes, which recommendations did you make?

Int J Sports Med | DOI 10.1055/a-2684-8925 | © Thieme. All rights reserved.

6. Other
Based on the information gathered during the After Career Consultation (medical history, online form, examination), are there any recommendations provided to the player with regard to other health aspects?

- Yes
- No

If yes, how would you rate these recommendations?

- Slightly significant
- Significant
- Very significant

If yes, which recommendations did you make?

7. Based on your assessment, do you plan to arrange any further investigation(s) for the retired footballer?

- Yes
- No

If yes, which investigation(s)?

8. Do you plan to arrange follow-up for the retired footballer?

- Yes
- No

If yes, when?

9. Do you plan to arrange secondary referral for the retired footballer?

- Yes
- No

If yes, to which specialty will you refer the player to?

Int J Sports Med | DOI 10.1055/a-2684-8925 | © Thieme. All rights reserved.

10. Provided the player has consented to it, do you plan to share the report from the After Career Consultation with the retired footballer's Primary Care Physician?

- Yes
- No

Int J Sports Med | DOI 10.1055/a-2684-8925 | © Thieme. All rights reserved.
